# Assessment of Foetal Exposure to the Homogeneous Magnetic Field Harmonic Spectrum Generated by Electricity Transmission and Distribution Networks

**DOI:** 10.3390/ijerph120403667

**Published:** 2015-04-01

**Authors:** Serena Fiocchi, Ilaria Liorni, Marta Parazzini, Paolo Ravazzani

**Affiliations:** 1 Istituto di Elettronica e di Ingegneria dell’Informazione e delle Telecomunicazioni IEIIT, CNR Consiglio Nazionale delle Ricerche, Piazza Leonardo da Vinci 32, Milan 20133, Italy; E-Mails: ilaria.liorni@ieiit.cnr.it (I.L.); marta.parazzini@ieiit.cnr.it (M.P.); paolo.ravazzani@ieiit.cnr.it (P.R.); 2 Dipartimento di Elettronica, Informazione e Bioingegneria DEIB, Politecnico di Milano, Piazza Leonardo da Vinci 32, Milan 20133, Italy

**Keywords:** ELF-EMF, foetus exposure, harmonics, power lines

## Abstract

During the last decades studies addressing the effects of exposure to Extremely Low Frequency Electromagnetic Fields (ELF-EMF) have pointed out a possible link between those fields emitted by power lines and childhood leukaemia. They have also stressed the importance of also including in the assessment the contribution of frequency components, namely harmonics, other than the fundamental one. Based on the spectrum of supply voltage networks allowed by the European standard for electricity quality assessment, in this study the exposure of high-resolution three-dimensional models of foetuses to the whole harmonic content of a uniform magnetic field with a fundamental frequency of 50 Hz, was assessed. The results show that the main contribution in terms of induced electric fields to the foetal exposure is given by the fundamental frequency component. The harmonic components add some contributions to the overall level of electric fields, however, due to the extremely low permitted amplitude of the harmonic components with respect to the fundamental, their amplitudes are low. The level of the induced electric field is also much lower than the limits suggested by the guidelines for general public exposure, when the amplitude of the incident magnetic field is set at the maximum permitted level.

## 1. Introduction

It is well known and widely accepted that exposure to Extremely Low Frequency (ELF) electromagnetic fields (EMF) causes well-defined acute biological effects on the nervous system, which include in particular nerve stimulation as well as the induction of retinal phospenes [1,2,3,4]. Furthermore, at the late 1970s, Wertheimer and Leeper [5] indicated a possible association between long-term exposure to ELF-EMF and an increased risk of childhood cancer. Since then, many expert-group reports, as well as independent epidemiological studies have specifically addressed this issue and in particular the relationship between the exposure to ELF-EMF produced by transmission lines and childhood leukaemia (see e.g., [6,7,8,9,10,11,12,13,14,15,16,17,18,19]). All this great amount of studies has been mainly based on the investigation of the exposure from electricity transmission and distribution networks at 50 Hz (or 60 Hz in some countries; in the rest of this Introduction, the term 50 Hz should be read as either 50 or 60 Hz, as a function of the country considered). The overall conclusion has been that the exposure to 50 Hz magnetic fields could be associated to an increased risk of leukaemia in children. This has brought the International Agency for Research on Cancer (IARC) [20] to classify ELF magnetic fields as “possibly carcinogenic to humans” in 2002. Since then, many studies have investigated the assessment of the exposure to magnetic field at the specific frequency of 50 Hz.

However, many authors have stressed the possible influence on ELF health effects of frequency components other than the fundamental 50 Hz one (see e.g., [21,22,23,24,25,26,27]). From that perspective, one should consider that the sources generating magnetic fields, in addition to the fundamental sinusoidal component at 50 Hz, are emitting additional frequency components, which can be analysed by the so-called sinusoidal harmonics, mainly due to non-linear electric loads. They can be defined as sinusoidal components of a signal, each of them at a frequency of an integer number of times the fundamental frequency. Day after day, this is becoming increasingly important, considering that the sources of distortion (*i.e.*, causes of introduction of harmonics) are spreading due to the development of new technologies, which use non-linear loads, in common electrical appliances and industrial equipment. This spread, besides impairing the quality of the supplied electrical power and producing damage to the devices, can increase consistently the amount of delivered power and thus increase the levels of exposure in an uncontrolled way [28].

So far, the available studies addressing the evaluation of the ELF-EMF exposure assessment at tissue level have been focused only on the exposure to 50 Hz magnetic fields of children, adults, women and pregnant women (see e.g., the studies of [29,30,31,32,33]). However, no study has addressed the estimation of the exposure at tissue level considering the contribution of the harmonics, least of all on foetuses.

The aim of this study was therefore to assess this latter exposure considering the harmonic content of a uniform magnetic field at 50 Hz. It will be performed on high-resolution three-dimensional models of pregnant women at 3, 7, 9 months gestational age. The harmonics considered here are based on the spectrum of supply voltage networks, as described in the European technical standards for the quality assessment of electrical power.

## 2. Material and Methods

### 2.1. Harmonic Spectrum

So far, only a few studies have dealt with the estimation of the harmonic components of a uniform magnetic field. The scarce available data (see e.g., [34,35]) does not fit the needs of the present study, in that they are highly specific in terms of sources and hence they cannot be used to build a realistic exposure scenario for a magnetic field composed by a fundamental component at 50 Hz plus harmonics. A possible solution could be to build a worst-case exposure scenario from the data provided by the available regulation addressing the quality of the power distribution. The presence of harmonics in the supply voltage signal can highly corrupt the signals delivered to the customer equipment and consequently cause damage to them. To prevent this situation, quality standards providing specific limits and tolerances that define the minimum requirements of the characteristics of electricity supplied by the distribution networks, were developed. In that regard, the European standard EN 50160 [36] “Voltage characteristics of electricity supplied by public distribution systems”, indicating the quality voltage parameters of electrical energy in public distribution systems, provides a basis for limiting and judgeing the severity of the contribution of harmonics, technically referred to as “harmonic distortion”. Its general approach is to express the supply characteristics of a distribution network by reference to the nominal voltage or declared voltage, as appropriate. Therefore, the maximum allowed amplitude of a voltage harmonic component is indicated as a percentage of the amplitude of the fundamental component (*i.e.*, at 50 Hz in Europe). To that purpose, the networks are clustered into three classes, as a function of the root mean square (rms) value of the supply voltage of the fundamental component: Low Voltage (LV), Medium Voltage (MV) and High-Voltage (HV). Quantitatively, EN 50160 [36] limits the amplitude of the harmonics to be in a range from 0.5% to 6.0% (5.0% for HV networks) of the amplitude voltage of the fundamental component, as a function of the order of the harmonic (up to the twenty-fifth).

In this study, as worst-case exposure scenario, the amplitude over time of the public transmission and distribution networks voltage is set as the time summation of the fundamental component to all the harmonic components up to the twenty-fifth, each one set at the maximum percentage of the fundamental permitted by the standard. Considering the need to keep the time cost of the computational procedure reasonable (see below) and that extremely low amplitude components are expected to minimally contribute to the exposure, harmonics mandated to be of maximum amplitude lower than the 3% of the fundamental (2.5% for HV) have been neglected in our analysis. Table 1 shows therefore the harmonics components considered in our exposure scenario with their corresponding relative amplitudes with respect to the fundamental component.

**Table 1:** Harmonic component considered in the magnetic field exposure scenario and their amplitude in terms of percentage of the amplitude of the fundamental component, as provided by the European standard EN 50160.

Order h	Frequency [Hz]	LV&MV Relative Amplitude	HV Relative Amplitude
3	150	5.0%	3.0%
5	250	6.0%	5.0%
7	350	5.0%	4.0%
11	550	3.5%	3.0%
13	650	3.0%	2.5%

### 2.2. Computational Modelling

Simulations of the exposure to a magnetic field as described in the section above, have been conducted with the Magneto Quasi-Static low frequency solver of the simulation platform SEMCAD X v. 14.8.4 (by SPEAG Schmid & Partner Engineering AG, Zurich, Switzerland) [37], based on the scalar potential finite element (SPFE) method. In the low frequency range, where the maximum dimension of the computational domain is much smaller than the free space wavelength, the magnetic vector potential ***A***, ($\nabla \text{ x }A\;=\;\frac{\partial B}{dt}$), and the electric field ***E*** are decoupled. Thus, ***A*** can be calculated by solving the Biot-Savart law and ***E***, since the displacement current in the human body can be neglected with respect to the conduction current (σ >> jωε where σ and ε are the electrical conductivity and the permittivity of the tissues, respectively, and ω is the angular frequency of the field), can be calculated from the scalar potential φ by:

(1) −∇ ∙ σ∇*φ* = jω∇・(σA)

The exposure of three anatomical models of pregnant women and corresponding foetuses at 3, 7 and 9 months of Gestational Age (GA), has been simulated here. Those models were obtained from insertion of the corresponding models of foetus in the “non-pregnant” woman model “Ella” of the Virtual Family [38], partially deformed to account for the change in morphology due to pregnancy stages. A detailed description of the development of the pregnant women and foetuses models can be found in [39]. Up to 77 different tissues have been distinguished in the woman model and up to 15, 17 and 26 different tissues can be distinguished in the models of the foetuses at 3, 7 and 9 months GA, respectively. The number of tissues differs with GA because of the different stages of maturation of the organs.

**Figure 1:**
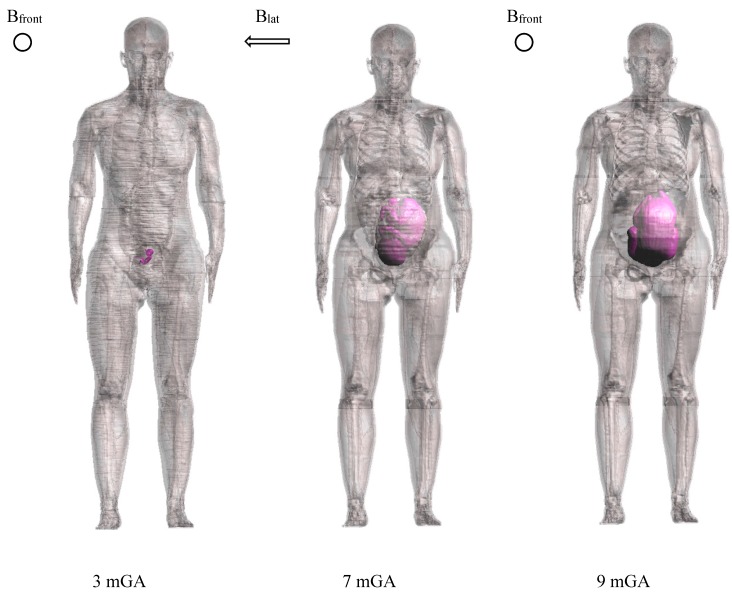
Exposure scenarios representation of the three pregnant woman models at 3, 7 and 9 months GA according to [33].

The orientation of the uniform magnetic field ***B*** and the foetal position (Figure 1) have been chosen based on the results of the study of Liorni and colleagues [33] on the exposure of pregnant women to 50 Hz magnetic fields. Hence, these field characteristics have been set to the same that induces the highest electric field ***E*** (expressed as the 99th percentile, E_99th_) in the bone tissue (identified as the target tissue for ELF magnetic field exposure, considering the possible association of that exposure and the increased risk of children leukaemia) and in the whole body of foetus, for each GA. In details:

- B_front_, for the 3 months GA (mGA) foetus;
- B_lat_, for the 7 mGA foetus;
- B_front_, for 9 mGA foetus.

where B_front_, B_lat_, and B_top_ means front-to-back, lateral (left-to-right) and top-to-bottom exposure, respectively. In the case of the 3 mGA foetus, the posture that induces the highest electric field ***E*** in the bone tissue and in the whole body of foetus corresponds to the foetus with the head up [33].

The dielectric properties were set according to the frequency of the harmonic component under simulation. For the fundamental component at 50 Hz, the dielectric properties have been set as in [33]. Since that approach included properties taken from the Hasgall *et al.* [40] database, whose validity was assured only up to 120 Hz, for the higher harmonic components the dielectric properties have been set on the basis of the classical Cole-Cole model [41,42,43,44] with the exception of the following tissues:

- The skin conductivity was set to 0.1 S/m for all frequencies to take into account the higher conductivity of deeper granular tissue, *i.e.*, dermis [45].
- Placenta and umbilical cord were modelled as blood because of their high perfusion [39].
- The conductivity of the amniotic fluid was set to 1.28 S/m, 1.27 S/m and 1.10 S/m at 3, 7 and 9 months GA, respectively, and taken constant across all frequencies [29,46].
- An average value between grey matter and white matter conductivities was assigned to medulla oblongata, pons and midbrain tissues (as in [33]).
- The lowest conductivity among the ones of the woman’s brain tissues was assigned to the foetal brain from a conservative point of view, to obtain higher electrical conductivity contrast with the embedding cerebrospinal fluid (CSF) (as in [33])
- The foetal bone conductivity was set to 0.35 S/m as in [33] taken as a weighted average of bone and blood (to take into consideration the infiltration of bone marrow at a very young age). It was also kept constant across all frequencies because of the relative stability of bone and blood conductivities values within the maximum harmonic frequency (*i.e.*, 650 Hz).
- Similarly, fat conductivity was set at 0.12 S/m, estimated as a blend between fat and muscle conductivities, according to the suggestion of Peyman *et al.* [47], related to the higher water content and blood presence at very young age.

### 2.3. Exposure Assessment

The exposure assessment was based on the estimation of the electric field induced both in the whole-body foetus and in each foetal tissue taken separately. In particular, the root mean square (*rms*) of the electric field averaged over cubes of 2 × 2 × 2 mm^3^ was calculated, as suggested by the ICNIRP exposure guidelines [4]. In detail, if 2 × 2 × 2 mm^3^ of contiguous tissue cannot be found (e.g., in the skin and in the retina and in many foetal tissues), the averaging has been performed over a smaller volume that includes only the specific tissue under analysis. The analysis was then accomplished by extracting the peak, the 99th and the 50th percentile (E_peak_, E_99th_ and E_50th_, respectively) of the E distribution of each tissue. The whole body foetus E_peak_, E_99th_ and E_50th_ are here considered as the maxima E_peak_, E_99th_ and E_50th_ found over all the tissues.

The vector potential ***A*** and the magnetic flux density ***B*** have been set to obtain a perfectly homogeneous magnetic field for all the considered polarizations in all the computational domain. Hence, the pregnant women have been exposed to uniform magnetic fields, whose peak amplitudes, for both the fundamental and harmonic components, were set to 1 μT. Considering the linearity of the problem, it is possible to scale the results to estimate the induced electric fields for any level of magnetic field actually used in the scenario under investigation.

The exposure of a pregnant woman to a magnetic field with a complex time content has been also evaluated in terms of its compliance with the current ICNIRP guidelines [4]. However, those guidelines refer to this specific condition only including in the Glossary “Pregnant Workers” in the “General Public” item, as an example of vulnerable groups or individuals. Neither specific action nor specific limits are suggested for these vulnerable individuals, and, least of all, for the foetus. This could be interpreted as to move to the assessor the decision of the best actions to be taken to protect these susceptible individuals. And hence to implicitly allow the assessor to implement a precise risk assessment plan, taking the decision about the conditions to be assessed in specific tissues other than those ones considered for the evaluation of the compliance of the woman. For that reason, it was decided here to consider the levels of exposure of the foetal tissues in the assessment of the compliance, comparing those levels with the ICNIRP guidelines [4] for general public exposure. They are shown in Table 2 as Reference Levels for magnetic flux density B and Basic Restrictions for the induced E_99th_, at each frequency considered in this study.

**Table 2:** Reference levels for magnetic flux density B_rms_ (2nd column) and Basic Restrictions for induced Electric field E_99th_ strength over the central nervous system CNS (brain and spinal cord) (3rd column) and over all the other tissues (4th column), as a function of the frequency (1st column), according to the ICNIRP Guidelines [4]. All values are rms.

Frequency (Hz)	Reference Level	Basic Restrictions	
	B (μT)	E_CNS_ (mV/m)	E_othertissues_ (mV/m)
50	200	20	400
150	200	60	400
250	200	100	400
350	200	140	400
550	145	220	400
650	123	260	400

To that purpose, and according to the guidelines for exposure to multiple frequency, the worst-case scenario has been obtained by setting the non-perturbed magnetic flux density *B* at the reference levels for each frequency considered. That means that:

(2) $$\overset{650\;Hz}{\underset{j\;=\;50\;Hz}{\sum }}\frac{B_{j}}{B_{R,j}}\;=\;1$$

where *B_j_* (μT) is the magnetic flux density at the frequency j, and *B_R,j_* (μT) is the reference level of the magnetic flux density at the same frequency j. Considering that the relationship between the amplitude of B_50Hz_ and the one of the other harmonics is based on the relative percentage as shown in Table 1, the maximum permitted amplitude of B_50Hz_ can be easily computed by Equation (2). The expanded equation becomes:

(3) $$\frac{B_{50}}{200}+\frac{B_{150}}{200}+\;\frac{B_{250}}{200}+\;\frac{B_{350}}{200}+\frac{B_{550}}{145}+\frac{B_{650}}{123}\;=\;1$$

where *B_j_* is calculated taking into account the relative percentage of the harmonics content with respect to the fundamental (*i.e.*, 50 Hz) as allowed by the EN 50160 (Table 1). This is true at a first approximation if we assume the proportionality between voltage and magnetic field amplitude, which is equivalent to assume the load constant, thus neglecting the impedance frequency-dependence.

Table 3 shows the results in terms of magnitude of each harmonic component for both the power supply networks considered. On a practical note, the results of the exposure assessment achieved with the amplitude of ***B*** set to 1 μT for all considered frequency component, have been scaled according to Table 3 and compared with the ICNIRP guidelines [4] (Table 2).

**Table 3:** Magnitude of the maxima magnetic flux density harmonic components applied in this study.

*B_j_*	LV&MV B_j_ [μT]	HV B_j_ [μT]
*B_50_*	159.1	166.4
*B_150_*	8.0	5.0
*B_250_*	9.5	8.3
*B_350_*	8.0	6.7
*B_550_*	5.6	5.0
*B_650_*	4.8	4.2

To estimate the difference from the induced electric field and the basic restrictions, one should scale the results in terms of E_99th_ found for peak *B_peak_* = 1 μT (*B_rms_* = 0.71) according to Table 3 and then by implementing the following equation:

(4) $$Max\;Fraction\;\%\;=\;\overset{650\;Hz}{\underset{j\;=\;50\;Hz}{\sum }}\frac{E_{j}}{E_{L,j}}$$

where *E_j_* (mV/m) is the internal electric field strength induced at frequency *j*, and *E_L,j_* is the induced electric field strength restriction at frequency *J.* (as reported in Table 2). The expanded equations (for CNS tissues and for all other tissues) become as follows:

  (5) $$\begin{array}{cc} \left[\text{CNS}\right] & Max\;Fraction\;\%\;=\;\frac{E_{50}}{20}+\frac{E_{150}}{60}+\;\frac{E_{250}}{100}+\;\frac{E_{350}}{140}+\frac{E_{550}}{220}+\frac{E_{650}}{260} \end{array}$$
  (6) $$\begin{array}{cc} \left[\text{Other}\;\text{tissues}\right] & Max\;Fraction\;\%\;=\;\frac{E_{50}}{400}+\frac{E_{150}}{400}+\;\frac{E_{250}}{400}+\;\frac{E_{350}}{400}+\frac{E_{550}}{400}+\frac{E_{650}}{400} \end{array}$$

In other words, those fractions indicate how far the results of this study are from the guidelines limits.

## 3. Results

Table 4 and Table 5 summarize the induced E_peak_, E_99th_ and E_50th_ fields in the whole-body foetus at 3, 7 and 9 months GA, for each frequency component, normalized to *B_peak,50Hz_* = 1 μT and scaled according to the relative amplitudes as provided by the EN 50160 standard (Table 1) for the two power supply networks considered. These results specifically provide a picture of the maximum levels of the foetal exposure to electricity transmission and distribution networks per μT of uniform magnetic field at 50 Hz. Therefore, once known the levels of magnetic field to which a foetus is exposed, by a simple scaling procedure, they can be used to assess the maxima realistic levels of exposure to electricity transmission and distribution networks.

The values reported were found in the fat (E_peak_ and E_99th_) and bladder (E_50th_), skin (E_peak_ and E_99th_) and liver (E_50th_ at 50 Hz) and brain (E_50th_ at harmonic frequencies) and skin (E_peak_ and E_99th_) and spinal cord (E_50th_ at all frequencies) for 3, 7, 9 months GA foetuses.

**Table 4:** Whole-body foetus induced electric field E_peak_, E_99th_ and E_50th_ at 3, 7 and 9 months GA, for each frequency, per μT of B_peak,50Hz,_ calculated according to the percentage provided by the standard EN 50160 in case of LV or MV electricity distribution networks exposure.

LV&MV	3 mGA			7 mGA			9 mGA		
Frequency (Hz)	E_peak_ (μV/m/μT)	E_99th_ (μV/m/μT)	E_50th_ (μV/m/μT)	E_peak_ (μV/m/μT)	E_99th_ (μV/m/μT)	E_50th_ (μV/m/μT)	E_peak_ (μV/m/μT)	E_99th_ (μV/m/μT)	E_50th_ (μV/m/μT)
50	19.3	14.2	9.0	40.6	27.6	10.8	56.9	38.6	16.5
150	2.3	1.4	1.0	5.5	3.6	1.6	7.4	4.7	2.2
250	4.6	2.7	2.0	10.7	7.0	3.1	14.7	9.4	4.5
350	5.3	3.1	2.3	12.4	8.2	3.6	17.2	10.9	5.2
550	5.8	3.4	2.5	13.6	8.9	3.9	18.9	12.0	5.7
650	5.9	3.4	2.6	13.8	9.0	4.0	19.2	12.2	5.8

**Table 5:** Whole-body foetus induced electric field E_peak_, E_99th_ and E_50th_ at 3, 7 and 9 months GA, for each frequency, per μT of B_peak,50Hz,_ calculated according to the percentage provided by the standard EN 50160 in case of HV electricity transmission networks exposure.

HV	3 mGA			7 mGA			9 mGA		
Frequency (Hz)	E_peak_ (μV/m/μT)	E_99th_ (μV/m/μT)	E_50th_ (μV/m/μT)	E_peak_ (μV/m/μT)	E_99th_ (μV/m/μT)	E_50th_ (μV/m/μT)	E_peak_ (μV/m/μT)	E_99th_ (μV/m/μT)	E_50th_ (μV/m/μT)
50	19.3	14.2	9.0	40.6	27.6	10.8	56.9	38.6	16.5
150	1.4	0.8	0.6	3.3	2.2	0.9	4.4	2.8	1.3
250	3.8	2.2	1.7	8.9	5.9	2.6	12.3	7.8	3.7
350	4.3	2.5	1.9	9.9	6.5	2.9	13.8	8.7	4.2
550	5.0	2.9	2.2	11.7	7.6	3.4	16.2	10.3	4.9
650	4.9	2.9	2.1	11.5	7.5	3.3	16.0	10.1	4.8

**Figure 2:**
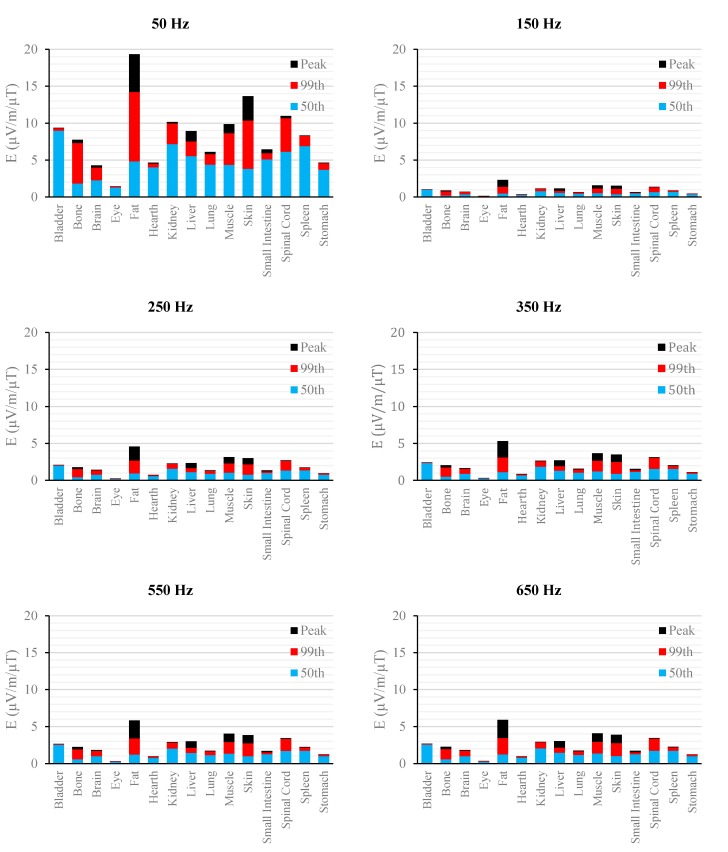
E (μV/m) per μT of 50 Hz B_peak_ induced in foetal tissues at 3 mGA for all the harmonic frequencies considering the standard EN 50160 for the LV&MV power supply networks. The bars indicate the E_50th_ (blue), the E_99th_ (red) percentiles and the E_peak_ (black) of each distribution.

**Figure 3:**
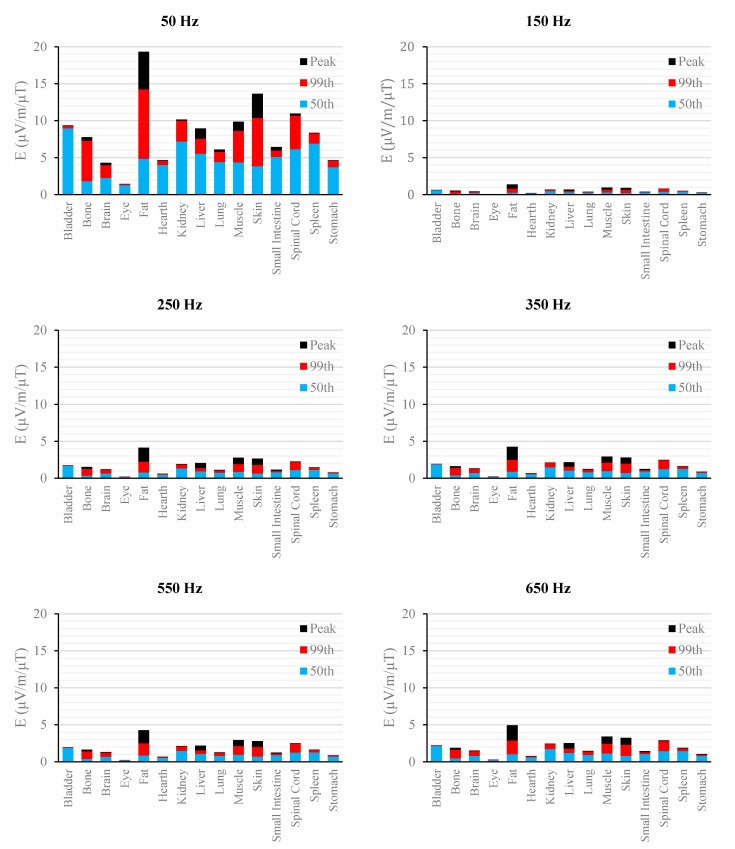
E (μV/m) per μT of 50 Hz B_peak_ induced in foetal tissues at 3 mGA for all the harmonic frequencies considering the standard EN 50160 for the HV power supply networks. The bars indicate the E_50th_ (blue), the E_99th_ (red) percentiles and the E_peak_ (black) of each distribution.

**Figure 4:**
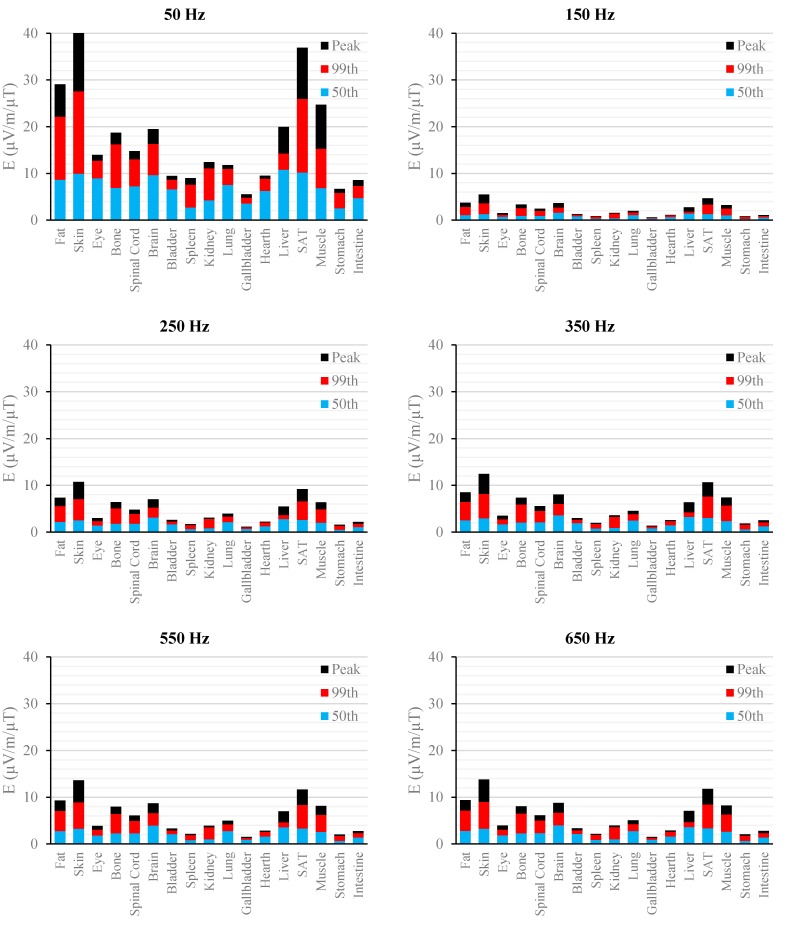
E (μV/m) per μT of 50 Hz B_peak_ induced in foetal tissues at 7 mGA for all the harmonic frequencies considering the standard EN 50160 for the LV&MV power supply networks. The bars indicate the E_50th_ (blue), the E_99th_ (red) percentiles and the E_peak_ (black) of each distribution.

**Figure 5:**
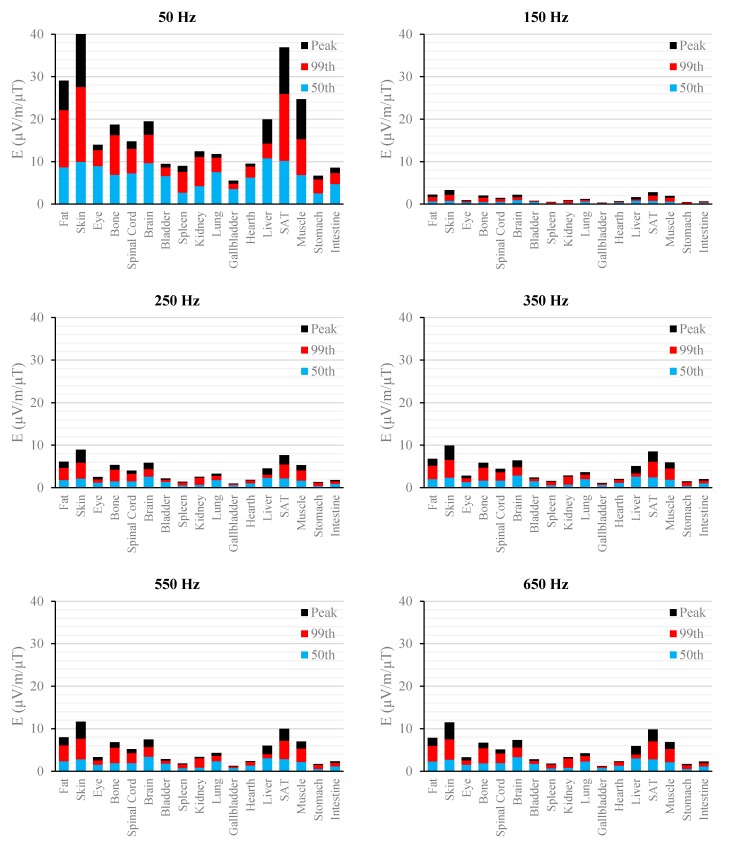
E (μV/m) per μT of 50 Hz B_peak_ induced in foetal tissues at 7 mGA for all the harmonic frequencies considering the standard EN 50160 for the HV power supply networks. The bars indicate the E_50th_ (blue), the E_99th_ (red) percentiles and the E_peak_ (black) of each distribution.

**Figure 6:**
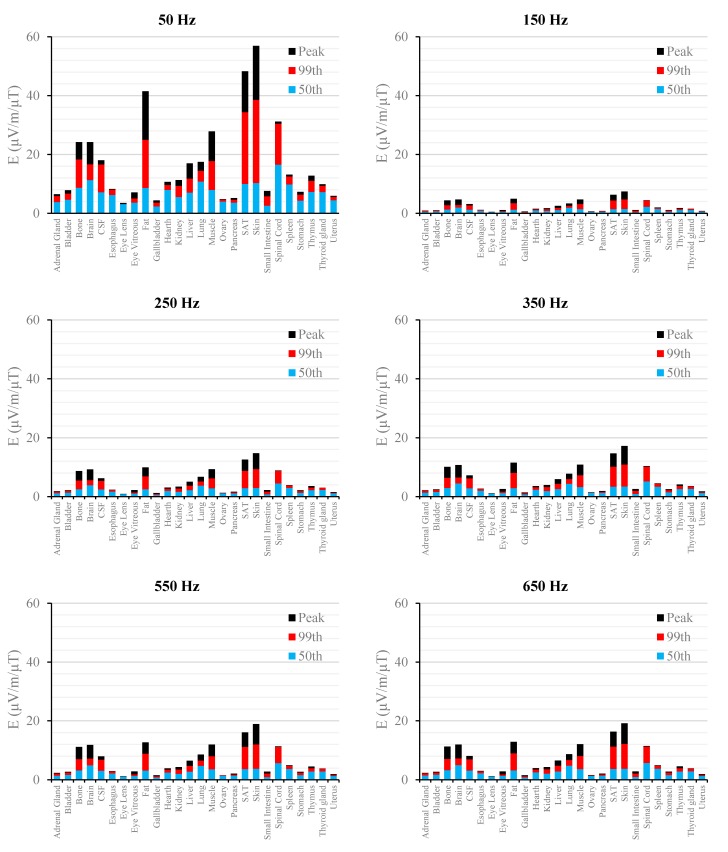
E (μV/m) per μT of 50 Hz B_peak_ induced in foetal tissues at 9 mGA for all the harmonic frequencies considering the standard EN 50160 for the LV&MV power supply networks. The bars indicate the E_50th_ (blue), the E_99th_ (red) percentiles and the E_peak_ (black) of each distribution.

**Figure 7:**
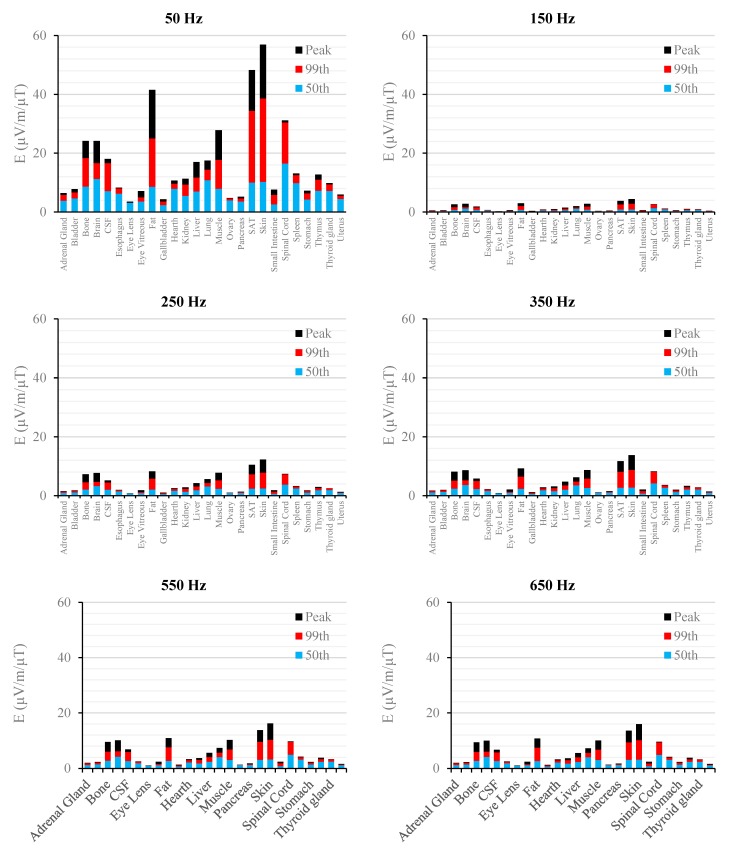
E (μV/m) per μT of 50 Hz B_peak_ induced in foetal tissues at 9 mGA for all the harmonic frequencies considering the standard EN 50160 for the HV power supply networks. The bars indicate the E_50th_ (blue), the E_99th_ (red) percentiles and the E_peak_ (black) of each distribution.

Figure 2, Figure 3, Figure 4, Figure 5, Figure 6 and Figure 7 show the calculated E_peak_, E_99th_ and E_50th_, for all harmonics considered in this study, over all tissues of the 3, 7 and 9 months GA foetuses, respectively, considering the exposure to *B_peak,50Hz_* = 1 μT for both the power supply networks considered. The maxima for each frequency coincide with the whole-body foetus values shown in Table 4.

**Figure 8:**
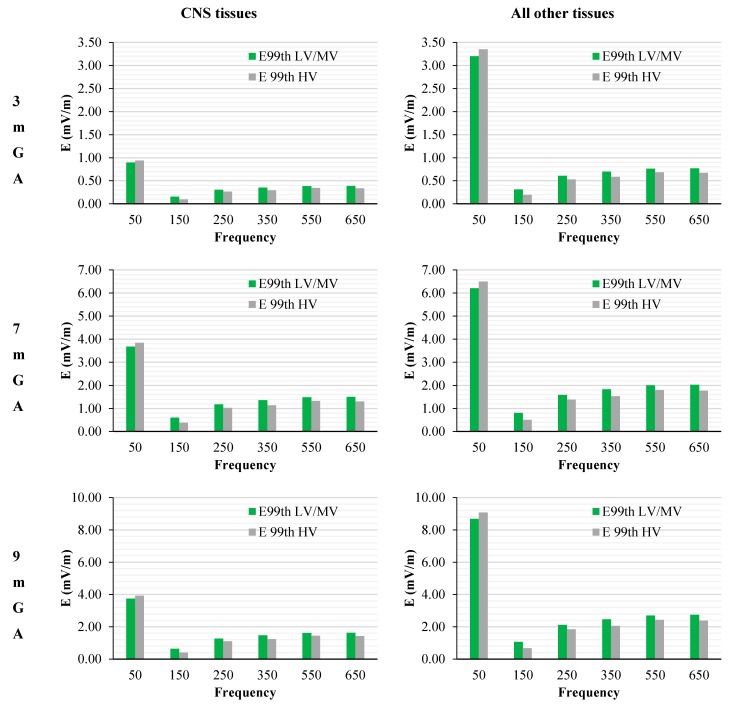
E_99th_ (mV/m) induced over CNS tissues (left) and all other tissues (right) at different GA as a function of the frequency of the component under investigation for the worst-case exposure (see the text for details). The green and grey bars indicate the two classes of networks identified by the EN 50160 standard, *i.e.*, Low and Medium Voltage (LV & MV) and the High Voltage (HV) networks, respectively.

The levels of the induced electric fields in the foetus were also compared with the exposure limits for general public exposure [4]. To that purpose, the maxima E_99th_ found among the CNS tissues over the head (*i.e.*, brain and eye according to the ICNIRP guidelines 2010 [4]) and among all the other tissues for each frequency have been scaled according to the values shown in Table 3, that identifies the worst-case exposure scenario. Figure 8 shows these data as a function of the frequency of the component under consideration and considering the standards for both the Low and Medium Voltage (LV & MV) and the High Voltage (HV) networks, respectively.

**Figure 9:**
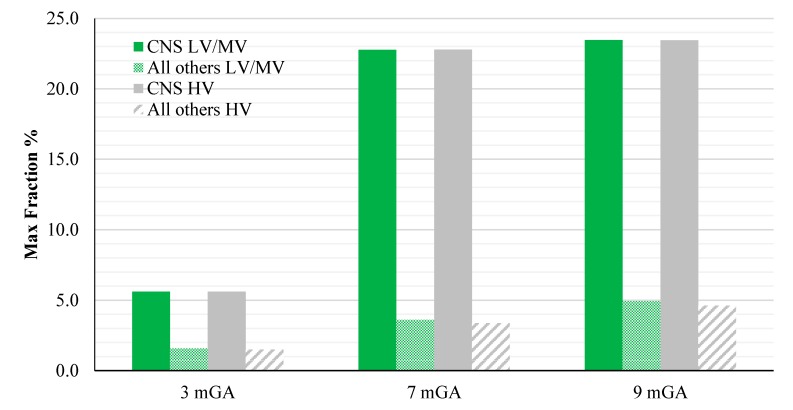
Maximum fraction of the ICNIRP Basic Restrictions for general public on the E_99th_ for the CNS and all other tissues, exposed to a magnetic field at the reference level using the standard EN 50160 (B levels shown in Table 4), at different GA.

The peak of the electric field is always at the fundamental frequency of 50 Hz and increases progressively with the stage of pregnancy. In particular, it ranges from 0.94 and 3.92 mV/m over CNS tissues of the head and from 3.35 to 9.1 mV/m over all other tissues, passing from 3 mGA to 9 mGA, respectively. The maximum E_99th_ over the CNS tissues, for all frequencies and across all the GAs were found in the brain at all the GA (16.7 μV/m/μT; 50 Hz, 9 mGA). The maximum induced E_99th_ over all other tissues were found over fat (14.2 μV/ m/μT; 650 Hz), skin (27.6 μV/m/μT; 50 Hz) and skin (38.6 μV/m/μT; 50 Hz) for 3 mGA, 7 mGA and 9 mGA, respectively. As expected, the electric field levels at the fundamental components due to the HV electricity transmission networks are higher than those ones due to the LV&MV electricity distribution networks, whereas an opposite behaviour has been found for the harmonic components. The comparison with the exposure standards was also analysed in terms of maximum fraction of the Basic Restrictions proposed by the ICNIRP guidelines [4], relative to both CNS tissues of the head and all the other tissues of the body, as described above. The results are summarized in Figure 9.

As one can see from this figure, all foetuses are exposed to levels far below the Basic Restrictions [4] for both CNS tissues of the head and all other tissues and for both the classes of voltage characteristics of the networks. The highest value is the one related to the CNS tissues, where a maximum of about 23.5 % (at 9 months GA) of the Basic Restrictions for both the classes of networks considered has been found. Lower values have been found in the other tissues, with a maximum fraction of only the 5%.

## 4. Discussion and Conclusions

The main outcome of this study is to provide a full picture of the EMF exposure of three realistic models of foetus at different gestational ages to electricity transmission and distribution networks. In particular, the source of EMF is here modelled taking into account the maxima spectral harmonic characteristics of the networks as provided by the quality standard EN 50160. It is important to note that the exposure scenario modelled in this study is worse than the one due to a realistic power supply network. Indeed, the few data available in literature ([34,48,49]) suggest that typically the prevailing harmonic is the fifth, with percentages ranging from 0.5 to 4.5% of the fundamental component. Moreover, the third and the seventh harmonics generally remain below 3% (see Table 1 for comparison with the percentage used in this study) and the Total Harmonic Distortion (THD—defined as the ratio of the root mean-square of the harmonic content to the root-mean square value of the fundamental quantity, expressed as a percent of the fundamental) below the 5%. On the contrary, in this study the THD resulted of the 10% and 8% for LV & MV and HV networks (using with the percentages of Table 1).

The first evidence coming from the analysis of the exposure to the full harmonic spectrum of the electricity transmission and distribution networks derives from the extremely low relative amplitude between the main component at 50 Hz and its harmonics (here ranging from 2.5% to 6.0%). Indeed, the induced electric fields due to the harmonic components are far lower than those ones induced by the main frequency at 50 Hz. As an example, in the foetal bone (one of the tissues considered of interest in leukaemia studies) and considering a source at the limit of the quality standard EN 50160 [36] (Figure 2, Figure 3, Figure 4, Figure 5, Figure 6 and Figure 7), the relative percentages of 99th and the 50th of the induced electric field at the harmonic frequencies with respect to the fundamental are summarized in Table 6.

**Table 6:** Relative percentage of the E_99th_ and E_50th_ in the foetal bone at the harmonic frequencies with respect to the same quantities calculated at 50 Hz, for 3, 7 and 9 months GA, and for both the supply networks, when the harmonic content at the source was set according to the percentage provided by the standard EN 50160.

E_bone, f_ /E_bone, 50_	3 mGA				7 mGA				9 mGA			
	LV & MV		HV		LV & MV		HV		LV & MV		HV	
**f (Hz)**	**99th ^(%)^**	**50th ^(%)^**	**99th ^(%)^**	**50th ^(%)^**	**99th ^(%)^**	**50th ^(%)^**	**99th ^(%)^**	**50th ^(%)^**	**99th ^(%)^**	**50th ^(%)^**	**99th ^(%)^**	**50th ^(%)^**
150	10.6	11.6	6.4	7.0	16.0	13.0	9.6	7.8	15.1	14.6	10.2	11.0
250	20.8	23.4	17.4	19.5	31.3	25.7	26.1	21.4	29.9	29.1	28.2	30.5
350	24.1	27.4	19.3	21.9	36.2	29.8	29.0	23.9	34.8	34.0	31.4	34.1
550	26.4	30.2	22.6	25.9	39.5	32.7	33.9	28.0	38.2	37.3	36.9	40.2
650	26.7	30.6	22.2	25.5	40.0	33.1	33.3	27.6	38.7	37.8	36.4	39.6

Again, this strong reduction of induced fields with frequencies can be also seen when the characteristics of the sources are set also considering the limit of the exposure levels (ICNIRP reference levels [4]) as shown in Figure 8, where, across the gestational ages of the foetus, the electric field shows a reduction of about the 55% (comparing the 50 Hz exposure with the 650 Hz) when the CNS tissues are considered, and of about the 70% (76% for 3mGA), when the other tissues are considered. These results are confirmed both when the LV&MV and HV electricity transmission and distribution networks are examined, even though this latter shows in all cases lower levels of induced E. This is due to the higher restrictions posed on HV power lines by the regulation of the quality of the electricity, as reviewed in Table 1.

As expected, the induced E increases with the gestational age. In more details, the average relative increase computed along the frequency of the E field in terms of E_peak_, E_99th_ and E_50th_, has been found of 2.3, 2.5 and 1.5, respectively passing from 3 to 7 months GA, and about 3.2, 3.4 and 2.2, respectively, passing from 3 to 9 months GA (Figure 2, Figure 3, Figure 4, Figure 5, Figure 6 and Figure 7).

As to the tissue dosimetry, the most exposed tissues (*i.e.*, tissue with the highest E_peak_, E_99th_), as expected, are also the most superficial ones for all GA and the frequencies considered (see in Figure 2, Figure 3 and Figure 4, skin, fat, SAT, when segmented, and muscle). On the contrary, the most exposed tissues on average (*i.e.*, with the highest E_50th_) strongly depend on the combination of foetal position and size and magnetic field polarization. At 3 months GA they are bladder, spinal cord, kidney and spleen, at 7 months GA brain, liver, skin and SAT, at 9 moths GA again brain, skin, SAT, lung and spinal cord. At 7 mGA, the maximum E_50th_ change position depending on frequency, passing from the liver (at 50 Hz) to the brain (at harmonic frequency); this is due the impact of the different choice of dielectric properties values (see Material an Methods section above) of these two tissues, which then affect mildly the whole electric field distribution. As for the whole-body electric fields, the levels of E_peak_, E_99th_ and E_50th_ show an increasing trend with the stage of pregnancy. Looking specifically at the bone tissue, the increase with foetal growth is even more conspicuous, suggesting that the foetuses close to the birth are exposed to larger induced electric fields. In particular, on average, along frequencies, it is equal to about 3.4, 3.2 and 4.0 for E_peak_, E_99th_ and E_50th_, respectively passing from 3 to 7 months GA, and about 4.6, 3.4 and 5.7 for E_peak_, E_99th_ and E_50th_, respectively, moving from 3 to 9 months GA.

In parallel to these considerations, an example on how these data can be concretely used in the comparison with the Basic Restrictions of the ICNIRP guidelines [4] was also given. The maximum fraction of 23.5% obtained considering together the fundamental component and the harmonics is similar with the one of the previous study [33] (maximum fraction of 23.6%), which only addressed the foetus exposure to 50 Hz homogenous magnetic fields. This similarity is quite expected considering that the larger contribution to the magnetic field is due to the 50 Hz frequency component and the contribution to the maximum fraction of the harmonic components is counterbalanced by the progressive reduction of the limits of the CNS Basic Restrictions with frequency, which implies that higher-order harmonic components are less weighted. Furthermore, in the interpretation of the maximum fraction (suggested by ICNIRP 2010 [4]), the phase relationship is not considered. This considerably overestimates the exposure, as shown by Leitgeb studies [50,51]. Those works found that many electric appliances emissions, characterized by complex frequency spectra of ELF magnetic field, exceeded ICNIRP reference levels up to almost two orders of magnitude. In these cases, and if the results here obtained will be used and therefore scaled to higher levels than the ICNIRP references, the existing ICNIRP spectral assessment rules are unnecessarily conservative and could lead to non-compliances. In those situations, alternative approaches should be adopted. The first alternative option to the spectral method would be to weight the external magnetic field and the induced electric field with a filter function which takes into account the fixed coherent phases angles of the field among harmonic frequencies, as suggested by [4] and [52]. Another approach, as suggested by [50], is to apply a nonlinear biology-based assessment rule, derived taking into account the nonlinearity of cellular membranes behaviour, which weights the higher (>3rd) harmonics not proportionally to frequency but with more appropriate contributions.

Finally yet importantly, the results of this study, as for any result obtained from computational assessment studies, are affected by a variable degree of uncertainty. Liorni and colleagues [33] discussed the uncertainty budget related to the estimation of the induced E in pregnant women exposed at 50 Hz. They found a maximum expanded uncertainty of 2.03 dB in foetal SAT for E_peak_ and 0.3 dB in foetal skin for E_99th._ Considering the linearity of E with frequency (up to 650 Hz) discussed above, the results of Liorni and colleagues apply also in this study.

Besides that, the major source of uncertainty linked to the results of this study is the representativeness of the pregnant woman models and the tissue parameters of the whole population included in the process of risk assessment.

To this purpose, the accuracy of the fetal anatomy was already discussed by [53] where some physiological and anatomical reference data of fetuses at different gestational ages were compared with the same data of the models used.

As to the dielectric properties, the main source of uncertainty is given by the intrinsic variability due to the tissue inhomogeneity and to the large difference in tissues structure and composition. The measurement of the dielectric properties of foetal tissues here used, have been performed by Gabriel and colleagues [54], who observed a variability ranging from 3 to 15%, with a peak of 25% for skin and skull tissues. Moreover they also stated that, in view of the large measurements performed per tissues, the total standard uncertainty is significantly lower than those percentages.

Other considerations and uncertainty assessment relative to the pregnant woman models are discussed in [33,39,53,55]. Even if these studies examined the uncertainty in the results of localized sources, the conclusions held also for the whole body exposure, since the uncertainty is certainly lower; this is due to the minor impact of dielectric properties and source and foetal position variability to the total budget uncertainty, as discussed in [39].

In conclusion, this study showed that in the exposure to the magnetic fields generated by electricity transmission and distribution networks, the harmonic components add some contributions to the overall level of the electric field induced in foetal tissues. However, although these contributions increase with frequency and cannot be neglected, their amplitude is low, due to the extremely low permitted amplitude of the harmonic components with respect to the fundamental one, even in the worst-case scenario here considered. The levels of the electric field in the foetus tissues are also extremely lower than the limits suggested by the ICNIRP 2010 guidelines [4] for general public exposure, when the amplitude of the incident magnetic field is set at the maximum permitted level by the guidelines themselves.
